# Use of TNF inhibitors in the treatment of PAPA syndrome

**DOI:** 10.1186/1546-0096-13-S1-P138

**Published:** 2015-09-28

**Authors:** D Stone, A Ombrello, A Almeida de Jesus, P Hoffmann, A Jones, R Goldbach-Mansky, K Barron, D Kastner

**Affiliations:** 1 NIH, Bethesda, USA

## Introduction

PAPA syndrome (pyogenic arthritis, pyoderma gangrenosum and acne syndrome) is a rare autoinflammatory disease caused by mutations in the *PSTPIP1* gene. This disease is difficult to treat, but the combination of prednisone, an IL-1 inhibitor and a TNF-inhibitor has, in our experience, helped even the most severely affected patients. Treatment with anakinra appears to prevent most of the severe joint manifestations, and treatment with a TNF-inhibitor is most effective for treating and preventing the pyoderma gangrenosum lesions.

## Objectives

We report our observations on the treatment of 4 severely affected PAPA syndrome patients with extensive pyoderma gangrenosum lesions.

## Patients and methods

Medical records from 4 patients with PAPA syndrome and severe pyoderma gangrenosum skin lesions were reviewed. Three of these patients had remained at our institution for extended visits while different therapeutic regimens were tried. Photos of lesions were taken periodically. CRP and ESR were followed.

## Results

One patient, an 8 year old girl with extensive lesions on her extremities requiring sedation for dressing changes, had been treated with adalimumab, 20 mg every 14 days, and pulses of methylprednisolone without effect. She showed some improvement after being treated with infliximab. However, she developed generalized urticaria after the second infusion. Despite etanercept, 2 mg/kg/week, and frequent infusions of methylprednisolone, she worsened and did not improve until she was started on golimumab, 50 mg every 10 days. She eventually healed completely.

A second teenage patient had extensive pyoderma gangrenosum lesions, severe acne, and elevated inflammatory markers. Etanercept was stopped when he developed elevated transaminases. Adalimumab, 40 mg every week, led to a minimal improvement. Infliximab caused an anaphylactic reaction on the second infusion. Golimumab, 100 mg every 7 to 14 days, and a course of isotretinoin led to the greatest improvement in this patient.

Two other patients with difficult-to-treat PAPA disease improved, one on golimumab after an anaphylactic reaction to infliximab and the other on high doses of infliximab. Both had shown minimal improvement on etanercept and adalimumab.

## Conclusion

Our observations indicate that treatment with infliximab, if tolerated, or high dose golimumab may be more effective than high doses of etanercept or adalimumab in those patients with PAPA syndrome and severe pyoderma gangrenosum lesions.

